# Real-world outcomes and management trends in uncomplicated type B aortic dissection

**DOI:** 10.1093/icvts/ivaf089

**Published:** 2025-04-11

**Authors:** Chikara Ueki, Eiji Nakatani, Akira Sugawara

**Affiliations:** Graduate School of Public Health, Shizuoka Graduate University of Public Health, Shizuoka, Japan; Graduate School of Public Health, Shizuoka Graduate University of Public Health, Shizuoka, Japan; Department of Biostatistics and Health Data Science, Graduate School of Medical Science, Nagoya City University, Nagoya, Japan; Graduate School of Public Health, Shizuoka Graduate University of Public Health, Shizuoka, Japan; Department of Gastroenterology, Shizuoka General Hospital, Shizuoka, Japan

**Keywords:** type B aortic dissection, thoracic endovascular aortic repair, optimal medical management, real-world data

## Abstract

**OBJECTIVES:**

Uncomplicated type B aortic dissection (uTBAD) accounts for a significant proportion of TBAD cases, but large-scale data on their prognosis remain limited. This study aims to evaluate real-world management and outcomes of uTBAD.

**METHODS:**

Patients aged 20 or older admitted for acute TBAD between 1 April 2013 and 30 September 2020 were included in the analysis set. They were classified as uTBAD 1 month after admission. Data were sourced from the Shizuoka Kokuho Database, a regional claims database. The primary outcomes were all-cause mortality and aortic events (death, type A dissection, rupture or surgery). Cumulative event rates were estimated using the Kaplan–Meier method. Outcomes of patients treated with thoracic endovascular aortic repair (TEVAR) versus medical therapy were compared using inverse probability weighting.

**RESULTS:**

A total of 1292 uTBAD patients were identified. Sixty-seven patients underwent TEVAR within 12 months, with a cumulative TEVAR rate of 5.4%. The cumulative mortality was significantly higher in comparison to the age- and sex-adjusted general population (1 year: 15.0% vs 6.7%, 3 years: 28.6% vs 18.6%, *P* < 0.001). Aortic events occurred in 22.1%, 30.0% and 36.7% at 1, 2 and 3 years, respectively. TEVAR within 12 months was associated with a trend towards lower mortality (adjusted hazard ratio 0.53, 95% confidence interval 0.27–1.04) and fewer aortic events (adjusted hazard ratio 0.54, 95% confidence interval 0.29–1.01) compared to medical therapy.

**CONCLUSIONS:**

uTBAD patients have poorer survival and higher rates of aortic events compared to the general population. TEVAR within 12 months can potentially improve patient outcomes.

## Graphical Abstract

**Figure:**
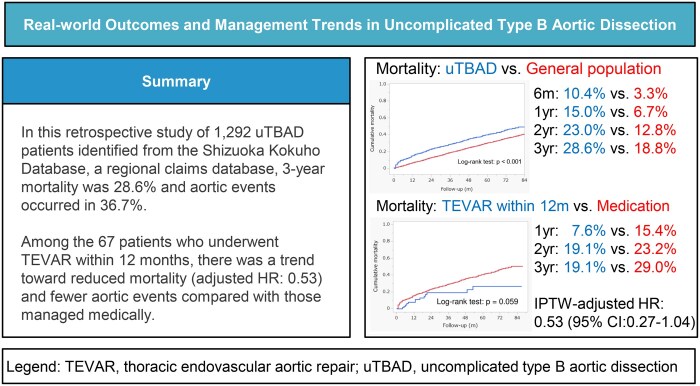

## INTRODUCTION

Type B aortic dissection (TBAD) is a life-threatening condition that constitutes a major portion of aortic pathologies. Approximately 75% of TBAD cases are classified as uncomplicated TBAD (uTBAD) [1]. For these patients, optimal medical therapy has been the standard of care, providing favourable short-term survival outcomes [2, 3]. However, despite initial clinical stability, many medically managed uTBAD patients develop long-term complications, including progressive aortic dilation, often requiring surgical intervention [4, 5]. To address these late complications, several randomized controlled trials have evaluated the efficacy of prophylactic thoracic endovascular aortic repair (TEVAR) [6–8]. These studies suggest that preventive TEVAR may improve long-term outcomes compared to optimal medical therapy by preventing late complications [6–8]. As a result, interest in preventive TEVAR to achieve better long-term prognosis in uTBAD patients has increased [3].

As evidence supporting preventive TEVAR for uTBAD continues to grow, there is an increasing need to update our understanding of the natural course of uTBAD in contemporary clinical practice. However, reports on the natural history of medically treated uTBAD remain limited [1, 4, 5]. A large 2013 report from the International Registry of Acute Aortic Dissection (IRAD) found that in-hospital mortality for medically managed TBAD patients was 8.7%, with a 1-year mortality rate of 9.8%. Despite these relatively favourable early outcomes, the study reported a high incidence of aortic dilation or new aneurysm formation at 5 years, affecting 73.3% of patients [9]. Although this study included 860 cases of medically managed TBAD, it had the limitation of including 37.3% of patients with complicated TBAD, potentially confounding the results [9]. Because uTBAD is a rare condition, traditional observational studies often struggle to enroll enough cases, making recent claims-based research and nationwide registry studies valuable in understanding the prognosis and long-term outcomes of these patients [10, 11].

This study aims to evaluate the prognosis and management of patients with uTBAD using large-scale claims data from the Shizuoka Kokuho Database (SKDB). This study provides large-scale data on the natural history of uTBAD, offering essential baseline information for future research and clinical trials.

## PATIENTS AND METHODS

### Ethical considerations

This study was conducted in accordance with the ethical principles outlined in the Declaration of Helsinki. The use of anonymized claims data for research purposes was approved by the ethics committee of Shizuoka General University Public Health (approval number SGUPH_2021_001_076) on 29 September 2023. Informed consent was waived due to the retrospective nature of the study and the use of anonymized data.

### Study design and data source

This study is a retrospective cohort analysis, conducted using data from the SKDB. The SKDB is a regional, population-based longitudinal cohort covering residents of Shizuoka Prefecture, Japan. The SKDB integrates individual-level data from health insurance claims, long-term care insurance claims and health checkup results. It covers residents enrolled in the National Health Insurance system, which serves individuals under 75 years of age, and the Latter-Stage Elderly Medical Care System for those aged 75 years and older. Data from 35 municipalities in Shizuoka Prefecture include hospitalizations, outpatient visits, prescribed medications, medical procedures and care services. The database contains patient demographics, ICD-10 diagnosis codes, treatment details, health checkup results and dates of death. All data were anonymized and linked using unique individual identifiers, enabling comprehensive tracking of patient outcomes [12].

The primary aim of this study was to describe the prognosis and management of patients with uTBAD. To complement this, we conducted an additional hypothesis-generating analysis to explore outcomes between two groups: patients who underwent TEVAR within the first 12 months and those who received medical therapy without TEVAR during the same period.

### Patients

**Figure 1:**
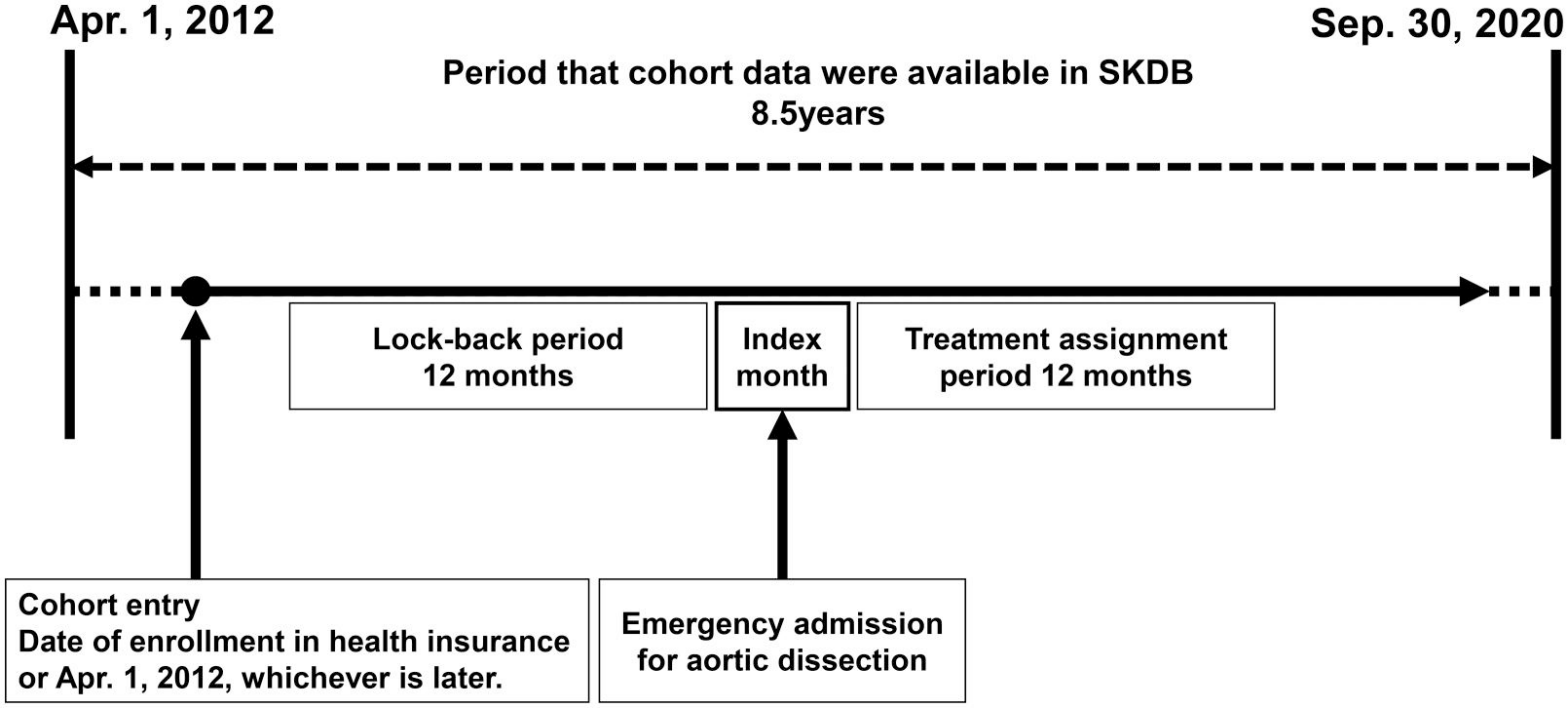
Study design. Cohort entry was defined as a patient’s date of registration with the health insurance provider or 1 April 2012, whichever occurred later. The index month was defined as the month in which emergency admission for aortic dissection was occurred. The lookback period was defined as the 12 months preceding the index month. Treatment assignment period was defined as the 12 months following the index month

Patients aged 20 or older were selected based on emergency admission for a primary diagnosis of aortic dissection (ICD-10 code I71.0) between 1 April 2013 and 30 September 2020 (Fig. 1). The SKDB contains data from 1 April 2012 to 30 September 2020. However, a 1-year baseline period was required to assess comorbidities and prior medical history. Therefore, patients admitted before 1 April 2013 were excluded to ensure a complete baseline period for analysis.

**Figure 2:**
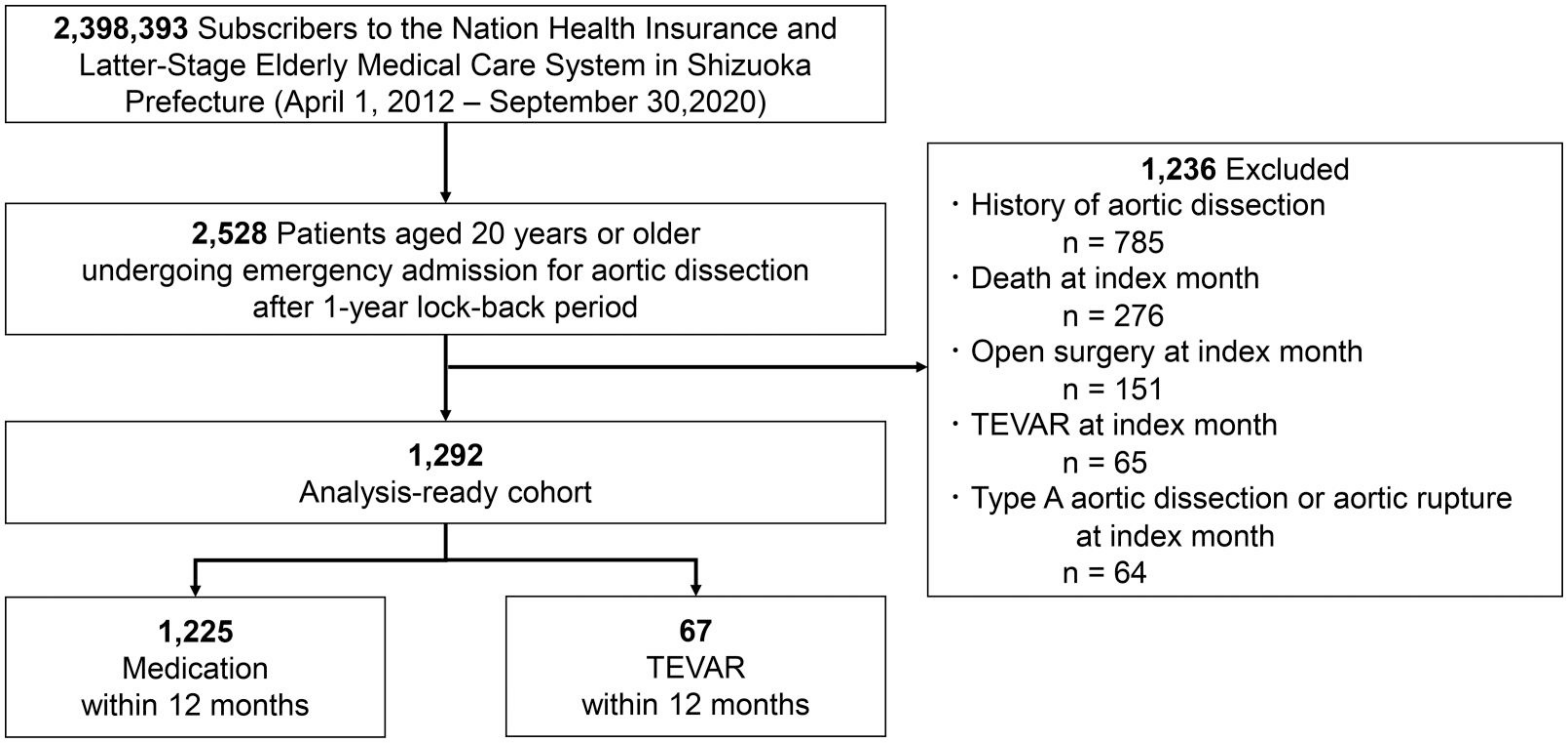
Flow diagram of study cohort enrollment

In our study, uTBAD was defined as patients who remained stable and free from complications by the end of the first month after admission. Patients who experienced death, surgical intervention (open surgery or TEVAR), type A aortic dissection or aortic rupture during the first month were excluded (Fig. 2). This 1-month timeframe was selected because the claims database used in this study provides data only at monthly intervals. Moreover, since the database lacks imaging findings and detailed surgical indications, this extended timeframe helps minimize the risk of including complicated cases in the cohort. Although our dataset does not allow us to directly identify limb or organ malperfusion, we anticipate that severe malperfusion typically results in early mortality or necessitates aortic surgical intervention. Thus, by excluding patients who died or underwent aortic surgical intervention within the first month, we have indirectly excluded the majority of severe malperfusion cases classified as complicated TBAD.

To ensure the accuracy of the cohort, the following exclusion criteria were applied:

1. Patients without a full 1-year baseline period before the index admission.
2. Patients with a previous diagnosis of aortic dissection in insurance claims prior to the index episode.
3. Patients who died during the month of admission.
4. Patients who underwent open surgery or TEVAR, as identified by procedure codes, during the month of admission.
5. Patients diagnosed with type A aortic dissection or aortic rupture during the month of admission.

Patients were categorized into two groups: those who underwent TEVAR within 12 months of their initial admission, and those who received medical therapy alone. A subgroup analysis was performed to compare outcomes between these two groups.

### Outcomes

The primary outcome of this study was all-cause mortality. Mortality was measured based on death records in the claims data. Secondary outcomes included aortic events, defined as a composite of death, surgical aortic interventions including both open surgery and TEVAR, the occurrence of type A aortic dissection and aortic rupture. All outcomes, except for mortality, were identified using ICD-10 diagnosis codes and procedural codes from the claims database.

In the additional analysis comparing TEVAR within the first 12 months to medical therapy, TEVAR performed within the first 12 months was classified as a protocol-defined intervention and was not treated as an aortic event. Aortic events were instead defined as a composite of additional TEVAR after 12 months, type A aortic dissection, aortic rupture or the need for further aortic interventions.

### Statistical analysis

Cumulative event rates and event-free survival were estimated using the Kaplan–Meier method. A control group from the general population was constructed to compare mortality rates. For each uTBAD patient, 100 age- and sex-matched individuals were randomly selected from the SKDB. This control group was used to compare all-cause mortality between uTBAD patients and the general population.

Inverse probability of treatment weighting (IPTW) based on propensity scores was applied to compare outcomes between patients who underwent TEVAR within 12 months and those who received medical therapy alone [13]. Propensity scores were calculated using a logistic regression model including the following variables: age, sex, region of residence, year of symptom onset, history of aortic aneurysm, history of TEVAR and the 25-item Elixhauser comorbidity index (shown in Table S2) [14]. The discrimination ability of the propensity score model was moderate, with an area under the curve of 0.739 (95% confidence interval [CI]: 0.681–0.798).

To assess robustness, sensitivity analyses were conducted to address IPTW outliers observed in the density plot. Trimming and filtering beyond the 99th percentile confirmed that extreme weights did not affect the main results (Table S1, Fig. S1) [13]. The balance of baseline covariates after IPTW adjustment was evaluated using standardized mean differences (SMDs). All covariates showed sufficient balance, with SMDs below or near the conventional threshold of 0.1, indicating successful weighting (Table S2, Fig. S2). Additionally, a sensitivity analysis excluding two low-frequency variables (hemiplegia and psychoses) with significant baseline imbalance yielded consistent results, further supporting the robustness of the IPTW-adjusted findings (Table S3).

All statistical analyses were performed using JMP Pro 17 (SAS Institute Inc., Cary, NC, USA, 1989–2023) and EZR (Saitama Medical Center, Jichi Medical University, Saitama, Japan) [15], a graphical user interface for R (The R Foundation for Statistical Computing, Vienna, Austria).

## RESULTS

### Patient background

A total of 1292 patients with a mean age of 77.3 ± 10.9 years were included in this study (Table 1). The cohort was 61.1% male, and the median follow-up period for the cohort was 29 months (range: 1–88 months).

**Table 1:** Patients’ characteristics

Variables	Overall cohort (*n* = 1292)	Medication (*n* = 1225)	TEVAR (*n* = 67)	*P* value
Male	789 (61.1)	741 (60.5)	48 (71.6)	0.063
Age (year)	77.4 ± 10.8	77.5 ± 10.9	74.5 ± 9.0	0.029
Age category				0.037
<50	29 (2.2)	28 (2.3)	1 (1.4)	
50–59	47 (3.6)	44 (3.6)	3 (4.5)	
60–69	204 (15.8)	190 (15.5)	14 (20.9)	
70–79	395 (30.6)	368 (30.0)	27 (40.3)	
80–89	491 (38.0)	470 (38.4)	21 (31.3)	
90–	126 (9.8)	125 (10.2)	1 (1.5)	
Year of admission				0.320
2013–2014	260 (20.1)	251 (20.5)	9 (13.4)	
2015–2016	369 (28.6)	351 (28.7)	18 (26.9)	
2017–2018	375 (29.0)	355 (29.0)	20 (29.9)	
2019–2020	288 (22.3)	268 (21.9)	20 (29.9)	
Area category				0.694
West	443 (34.3)	420 (34.3)	23 (34.3)	
Middle	421 (32.6)	401 (32.7)	20 (29.9)	
East	386 (29.9)	363 (29.6)	23 (34.3)	
Other	42 (3.3)	41 (3.4)	1 (1.5)	
Comorbidity				
Heart failure	287 (22.2)	276 (22.5)	11 (16.4)	0.225
Valvular disease	91 (7.0)	86 (7.0)	5 (7.5)	0.891
Arrhythmias	249 (19.3)	238 (19.4)	11 (16.4)	0.535
PAD	254 (19.7)	238 (19.4)	16 (23.9)	0.558
Pulmonary disease	329 (25.5)	309 (25.2)	20 (29.9)	0.405
Hypertension	791 (61.2)	750 (61.2)	41 (61.2)	0.996
Diabetes	69 (5.3)	66 (5.4)	3 (4.4)	0.870
Renal failure	120 (9.3)	110 (9.0)	10 (14.9)	0.128
Liver disease	184 (14.2)	175 (14.3)	9 (13.4)	0.845
Hemiplegia	25 (1.9)	25 (2.0)	0 (0.0)	0.238
Tumour	159 (12.3)	151 (12.3)	8 (11.9)	0.925
History of aortic aneurysm	150 (11.6)	138 (11.3)	12 (17.9)	0.120
Previous TEVAR	4 (0.3)	3 (0.2)	1 (1.5)	0.186
Follow-up (months)	27.5 (1–88)	29 (1–88)	22 (2–87)	0.557

Age is expressed as mean ± SD, and follow-up duration is expressed as median (IQR). Categorical variables are presented as *n* (%). *P* values represent the statistical significance of differences between the medication and TEVAR groups. PAD: peripheral arterial disease.

Patients were divided into two groups: 1225 received medical therapy, and 67 underwent TEVAR within 12 months of admission. The TEVAR group was slightly younger than the medical therapy group (74.5 ± 9.0 years vs 77.5 ± 10.9 years, *P* = 0.029). The proportion of men was higher in the TEVAR group than in the medical therapy group (71.6% vs 60.5%, *P* = 0.063), but the difference was not statistically significant. The median follow-up period was similar between the groups (TEVAR: 22 months, range: 2–87 months vs medical therapy: 29 months, range: 1–88 months, *P* = 0.557).

There was no significant difference in admission years between the two groups. However, TEVAR was more frequently performed in patients admitted in 2019–2020, while patients admitted in 2013–2014 were more likely to receive medical therapy.

### Primary outcome: mortality

**Figure 3:**
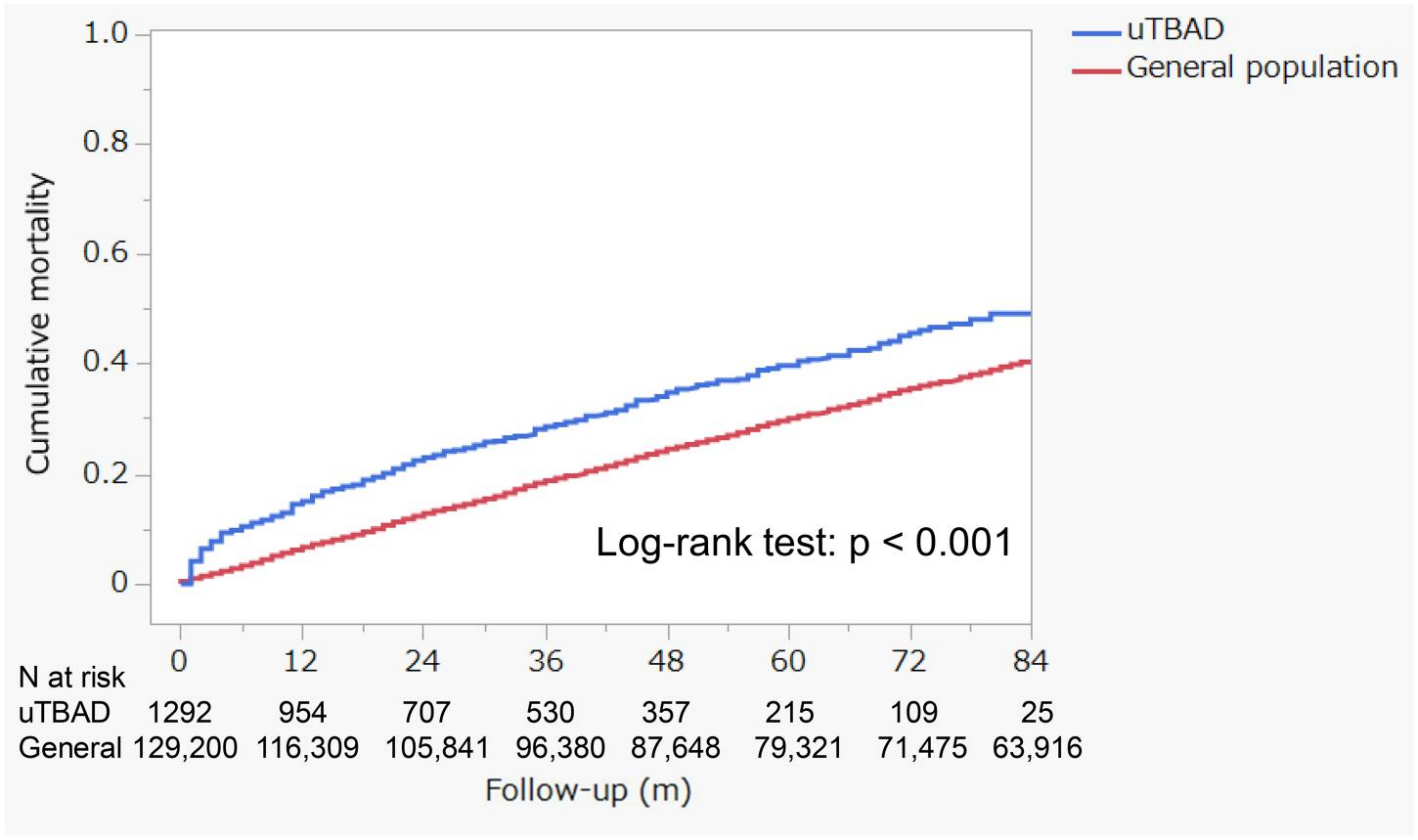
The cumulative all-cause mortality compares the uncomplicated type B aortic dissection cohort (*n* = 1292) with an age- and sex-matched general population cohort (*n* = 129 200), which consists of 100 individuals for each patient with uncomplicated type B aortic dissection

The cumulative mortality rates for the entire cohort were 15.0% at 1 year, 23.0% at 2 years and 28.6% at 3 years. These rates were significantly higher than those of the age- and sex-matched general population cohort (*n* = 129 200) from the same region, which had mortality rates of 6.7% at 1 year, 12.7% at 2 years and 18.6% at 3 years (Fig. 3, log-rank test: *P* < 0.001).

### Aortic events

**Figure 4:**
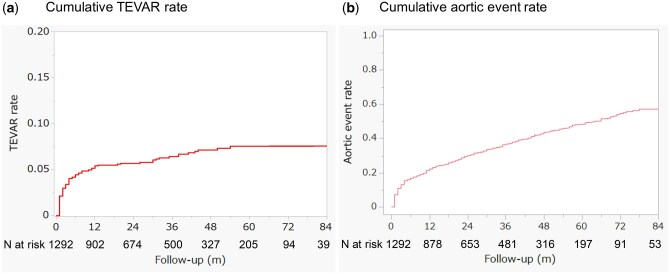
The cumulative rates of TEVAR (**a**) and aortic events (**b**) are presented for the entire uncomplicated type B aortic dissection cohort

In the overall cohort, 83 TEVAR procedures, 40 open aortic surgeries, 21 cases of type A aortic dissection and 19 cases of aortic rupture were observed after the first month of admission. Among these, within the first year after admission, there were 67 TEVAR procedures and 28 open aortic surgeries. The cumulative TEVAR rates were 3.4% at 3 months, 4.5% at 6 months, 5.4% at 1 year, 5.7% at 2 years and 6.4% at 3 years (Fig. 4a).

The cumulative incidence of aortic events, including death, aortic rupture, type A aortic dissection and the need for surgical intervention, was 16.8% at 6 months, 22.1% at 1 year, 30.0% at 2 years, and 36.7% at 3 years (Fig. 4b).

### Comparison between TEVAR and medical therapy

**Figure 5:**
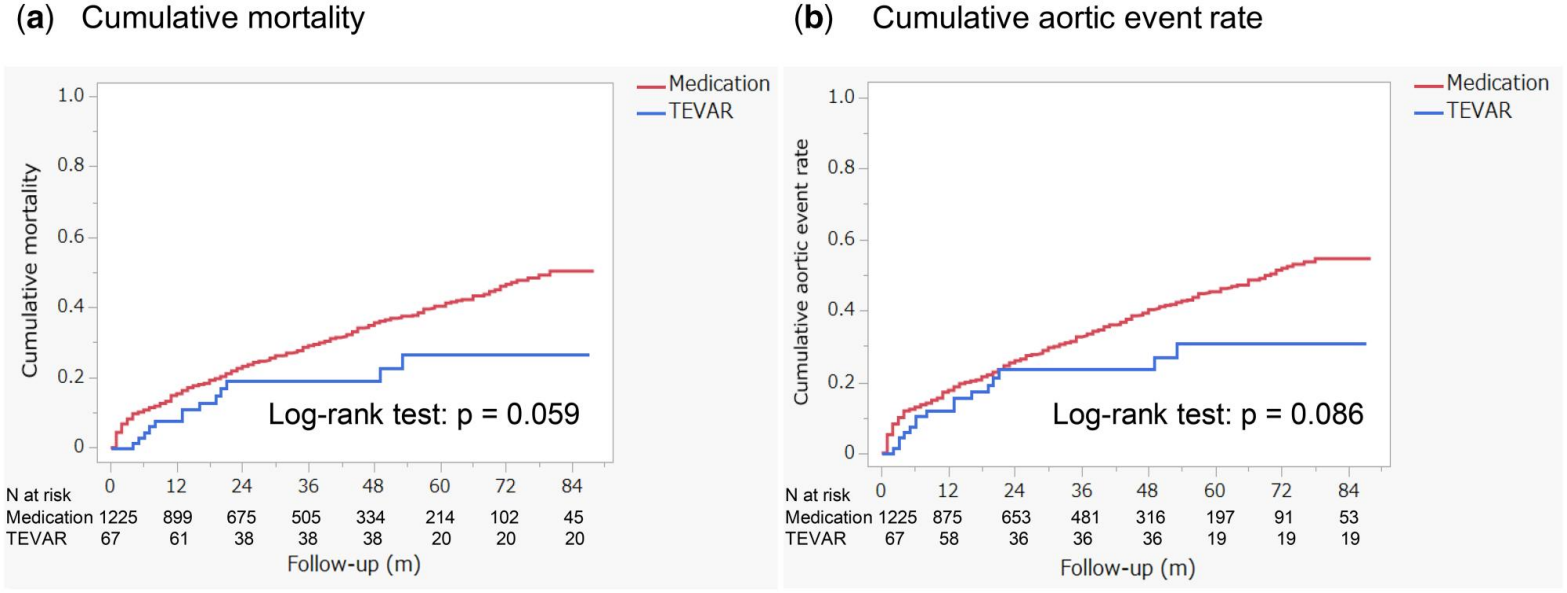
The comparison of all-cause mortality (**a**) and aortic event rates (**b**) between the TEVAR group and the medication group

In the crude analysis, the 1-year cumulative mortality rate was lower in the TEVAR group (7.6%) compared to the medical therapy group (15.4%). At 2 years, mortality rates were 19.1% in the TEVAR group and 23.2% in the medical therapy group. By 3 years, the rates were 19.1% for the TEVAR group and 29.0% for the medical therapy group. Although the mortality difference between the two groups did not reach statistical significance, there was a trend towards lower mortality in the TEVAR group (Fig. 5a, log-rank test: *P* = 0.059).

The cumulative incidence of aortic events was also lower in the TEVAR group. At 1 year, the rate was 12.1%, rising to 23.6% at 2 and 3 years. In contrast, the medical therapy group had a 17.7% incidence at 1 year, 26.0% at 2 years and 33.0% at 3 years (Fig. 5b, log-rank test: *P* = 0.086).

**Figure 6:**
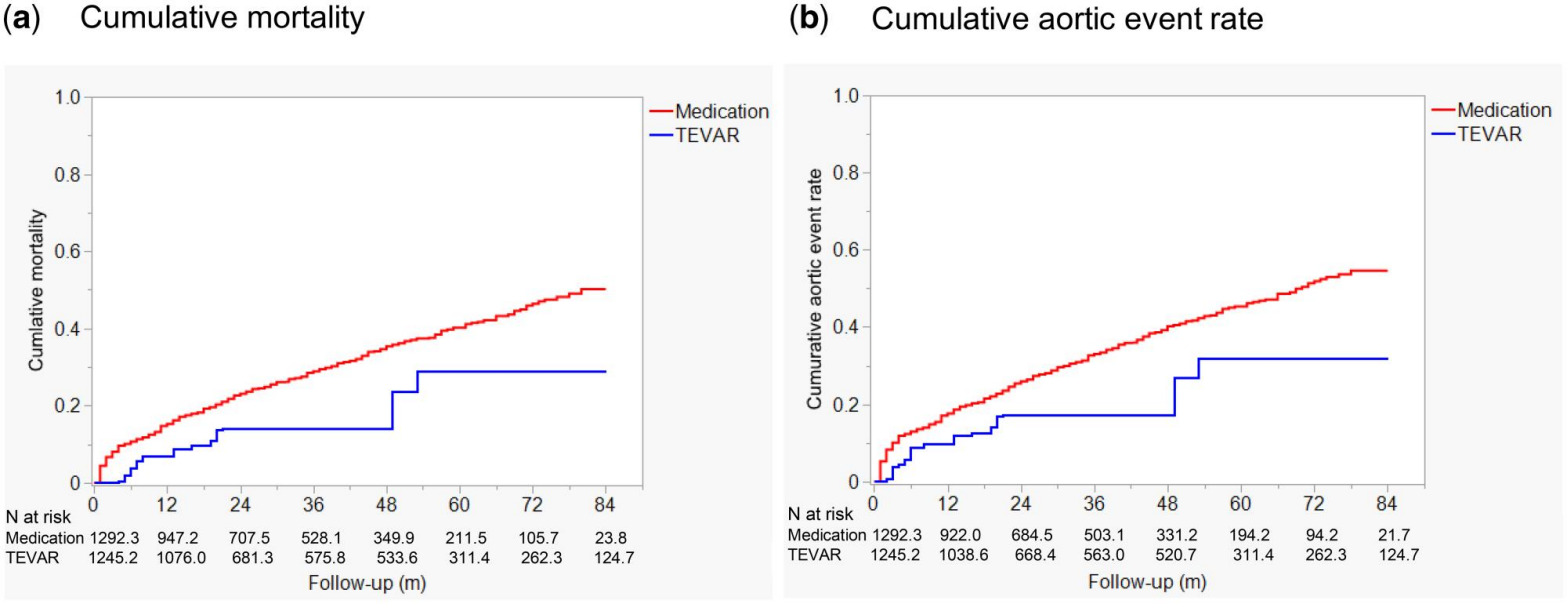
The comparison of cumulative all-cause mortality (**a**) and cumulative aortic event rates (**b**) between the TEVAR group and the medication group, adjusted using inverse probability of treatment weighting

After adjusting for baseline differences using IPTW, TEVAR showed a trend towards reduced mortality with an IPTW-adjusted hazard ratio (HR) of 0.53 [95% CI: 0.27–1.04, *P* = 0.063] (Fig. 6a). Similarly, the risk of aortic events was lower in the TEVAR group, with an IPTW-adjusted HR of 0.54 (95% CI: 0.29–1.01, *P* = 0.053), suggesting a trend towards reduced risk of aortic events in patients who underwent TEVAR (Fig. 6b). A sensitivity analysis was performed to assess the robustness of the results by excluding patients who underwent open aortic surgery within the first 12 months. After this exclusion, the adjusted HR for mortality in the TEVAR group compared to the medical therapy group was 0.54 (95% CI: 0.27–1.06, *P* = 0.071). For aortic events, the adjusted HR was 0.50 (95% CI: 0.25–0.98, *P* = 0.043).

## DISCUSSION

This study offers valuable insights into the management and prognosis of patients with uTBAD. Our results indicate that uTBAD patients have significantly higher cumulative mortality rates compared to the general population, highlighting the long-term risks associated with this condition. While TEVAR did not result in statistically significant reductions in mortality or aortic events, it showed a clear trend towards better outcomes compared to medical therapy alone. These findings suggest that more aggressive intervention strategies, such as TEVAR, can improve survival outcomes in uTBAD patients. However, further research is needed to confirm these potential benefits.

Several observational studies have examined the prognosis of patients with TBAD managed with medical therapy [4, 5, 9]. The largest cohort was reported by the IRAD registry, which included 860 medically treated patients (mean age 65.2 years, 61.8% male). The 5-year cumulative mortality rate in this group was 29.0%, highlighting the long-term risks of TBAD management [9]. However, this cohort also included approximately 37% of patients with complicated TBAD, limiting its applicability as a control group for contemporary outcomes in uTBAD patients [9].

Weissler and colleagues, using inpatient claims data from Medicare and Medicaid Services in the United States, reported high 5-year cumulative mortality rates for both the medically treated uTBAD group (49.0%) and the TEVAR group (44.5%) [10]. Both groups also had high rates of late aortic interventions (15.1% in the medical therapy group and 16.8% in the TEVAR group at 5 years) and aortic-related hospital admissions (30.7% and 34.7%, respectively) [10]. These complications likely contribute to the poor long-term survival seen in uTBAD patients.

Our study similarly found that overall survival in the uTBAD cohort was significantly worse than in the age- and sex-matched general population. Furthermore, the cumulative incidence of late aortic events was notably high, reaching 36.7% at 3 years. These findings underscore the ongoing risk of aortic complications, which likely contribute to the poor long-term prognosis in uTBAD patients, highlighting the need for improved management strategies.

In our cohort, only a small proportion of uTBAD patients underwent TEVAR within the first 12 months after symptom onset, with a cumulative rate of 5.4% at 1 year. Most TEVAR interventions occurred during the subacute phase, as indicated by cumulative rates of 3.4% at 3 months and 4.5% at 6 months. In comparison, Weissler *et al.* reported that 16% of uTBAD patients underwent TEVAR within 30 days, based on US Medicare and Medicaid claims data [10]. However, direct comparison is challenging due to differences in case definitions; our study excluded patients who received TEVAR within the first month, focusing on subacute cases. Overall, TEVAR use in real-world practice remains relatively low, consistent with surveys indicating that only about half of surgeons recommend TEVAR for uTBAD [16].

Despite the limited number of TEVAR cases in our study, patients who underwent TEVAR within 12 months showed a trend towards better outcomes. After adjusting for baseline characteristics using IPTW, TEVAR patients had lower HRs for both mortality (adjusted HR 0.53; 95% CI 0.27–1.04, *P* = 0.063) and aortic events (adjusted HR 0.54; 95% CI 0.29–1.01, *P* = 0.053). These results are consistent with previous studies, such as the INSTEAD-XL trial, which demonstrated that TEVAR can prevent aortic-related deaths and slow the progression of aortic disease in uTBAD patients [5, 7, 17, 18].

Based on our results, the selective use of preventive TEVAR during the subacute phase in patients with high-risk features may improve long-term outcomes in the whole uTBAD cohort. Although TEVAR has demonstrated potential benefits in improving survival and reducing aortic events, it has limitations. Proper patient selection is critical, as unnecessary interventions can lead to serious complications, including retrograde type A aortic dissection, stroke and paraplegia [9, 17–19]. This emphasizes the need to minimize unnecessary TEVAR procedures. TEVAR candidates should be selected based on high-risk features associated with poor outcomes from medical management alone. These features include patients presenting with high-risk imaging or clinical findings, such as an aortic diameter ≥40 mm, a false lumen diameter ≥22 mm, a primary entry size ≥10 mm, persistent pain or uncontrollable hypertension [2, 20–22]. However, these indicators are not always reliable. Thus, it is crucial to continuously monitor outcomes like mortality and aortic events across the entire uTBAD cohort, not just those who undergo interventions. A broader evaluation approach can offer valuable feedback to clinicians and policymakers, refining treatment strategies and patient selection criteria, and ultimately improving long-term outcomes for all uTBAD patients.

This study has several limitations. First, as with many cohort studies utilizing claims data, the lack of imaging findings and clinical decision-making details limits our ability to fully assess case complexity and the indications for TEVAR [10]. This limitation raises concerns, including the possibility that, even after excluding patients who experienced death or major aortic complications within the first month, some patients with radiographically defined complicated cases might have been included in the cohort. Additionally, preventive TEVAR may have been recorded as an aortic event in the database, potentially resulting in misclassification. Second, excluding patients who underwent TEVAR within the first month may have introduced selection bias by focusing only on subacute cases. Third, another limitation of this study is the advanced mean age of the cohort, which may affect the generalizability of the findings. Older patients might have reduced eligibility for TEVAR due to comorbidities or other clinical considerations, and the benefits of TEVAR in this population might be limited compared to younger patients. Finally, although IPTW was used to adjust for baseline differences, residual confounding from unmeasured variables cannot be completely excluded. Despite these limitations, our findings emphasize the need for comprehensive outcome assessments and careful patient selection to optimize uTBAD management. Further studies, particularly prospective trials, are necessary to confirm the benefits of TEVAR in this population and refine treatment strategies.

## CONCLUSIONS

Our findings highlight that uTBAD patients face significantly higher long-term mortality compared to the general population, with a cumulative incidence of aortic events reaching 36.7% at 3 years. Although TEVAR was performed in a small proportion of patients within 12 months, it showed a trend towards reducing mortality and aortic events. Our data offer essential reference points for evaluating the outcomes of TEVAR in uTBAD, providing a baseline against which future intervention strategies can be compared. Further research, particularly prospective trials, is necessary to confirm the benefits of preventive TEVAR in uTBAD patients and to refine treatment strategies.

## Supplementary Material

ivaf089_Supplementary_Data

## Data Availability

According to the terms of Shizuoka Prefecture’s data use agreement with local insurers, the analysed data cannot be provided to readers by the authors. Researchers interested in accessing this dataset may apply to Shizuoka Prefecture to request access. Please contact the staff of Shizuoka Graduate University of Public Health (e-mail: info@s-sph.ac.jp).

## Notes

Presented at the 38th EACTS Annual Meeting, Lisbon, Portugal, October 9–12, 2024.

## ABBREVIATIONS

CI: Confidence interval
HR: Hazard ratio
IPTW: Inverse probability of treatment weighting
IRAD: International Registry of Acute Aortic Dissection
SKDB: Shizuoka Kokuho Database
TBAD: Type B aortic dissection
TEVAR: Thoracic endovascular aortic repair
uTBAD: Uncomplicated type B aortic dissection
