# Supplementary material for: Real-world outcomes and management trends in uncomplicated type B aortic dissection
Source: Interdiscip Cardiovasc Thorac Surg. 2025 Apr 11;40(4):ivaf089. doi: 10.1093/icvts/ivaf089 (PMC12022216; doi:10.1093/icvts/ivaf089)
Supplement: ivaf089_Supplementary_Data [file ivaf089_supplementary_data.zip › ivaf089_Supplementary_Data/Supplementary Materials.docx]

**Supplementary Materials**

**Overview of Sensitivity Analyses**
**1. Handling Outliers in Inverse Probability of Treatment Weighting (IPTW)**

The distribution of inverse probability of treatment weights (IPTWs) was visualized using a density plot (Figure S1), which revealed the presence of outliers. To assess the impact of these extreme weights, we performed trimming and filtering beyond the 99th percentile (IPTW > 23.3). Table S1 summarizes the results of these analyses, showing that the adjusted hazard ratios (HRs) for mortality and aortic events remained consistent, confirming that the findings were not significantly influenced by these outliers.

**2. Evaluating Covariate Balance**

The standardized mean differences (SMDs) of baseline covariates before and after IPTW adjustment were assessed to evaluate the balance achieved through weighting. Table S2 demonstrates sufficient balance for most covariates in the main model, with the exception of the Area category (SMD = 0.109). Figure S2 further visualizes the improved balance after IPTW adjustment.

**3. Sensitivity Analysis Excluding Rare Variables**

To address potential bias from variables with no representation in the TEVAR group, a sensitivity analysis was conducted excluding two low-frequency variables (Hemiplegia and Psychoses). Table S3 compares the SMDs from the main model with those from this alternative model. The main IPTW-adjusted analysis yielded hazard ratios (HRs) of 0.53 (95% CI: 0.27–1.04, p=0.063) for mortality and 0.54 (95% CI: 0.29–1.01, p=0.053) for aortic events. In the sensitivity analysis excluding the two variables, the adjusted HRs were 0.51 (95% CI: 0.26–1.01, p=0.052) for mortality and 0.52 (95% CI: 0.28–0.98, p=0.044). These consistent findings confirm the robustness of the IPTW-adjusted results.

**Figure legends**

**Figure S1. Density plot of inverse probability weights (IPTWs) for the two treatment groups**:

The density plot illustrates the distribution of IPTWs on a logarithmic scale for the TEVAR group (blue) and the Medical group (red). Dots along the X-axis represent individual cases.

**Figure S2. Balance Plot: Standardized Mean Differences Before and After Adjustment**:

This plot displays the Standardized Mean Differences (SMD) for covariates before and after inverse probability of treatment weighting (IPTW). Crude SMD values are shown as blue circles, while IPTW-adjusted SMD values are displayed as red circles.

**Table S1. Sensitivity Analyses of Filtering and Trimming with 99th IPTW Percentile Threshold**

| Outcome | Analysis type | IPTW adjusted HR (95% CI) | p-value |
| --- | --- | --- | --- |
| Mortality | Main analysis | 0.53 (0.27 - 1.04) | 0.063 |
|  | Trimming | 0.62 (0.34 - 1.11) | 0.106 |
|  | Filtering | 0.53 (0.25 - 1.12) | 0.098 |
| Aortic event | Main analysis | 0.54 (0.29 - 1.01) | 0.053 |
|  | Trimming | 0.65 (0.38 - 1.12) | 0.119 |
|  | Filtering | 0.65 (0.34 - 1.24) | 0.189 |

This table presents the results of sensitivity analyses for mortality and aortic events using IPTW-adjusted HRs. Filtering was conducted by excluding cases exceeding the 99th IPTW percentile, while trimming applied weight capping at the same threshold. CI, confidence interval; HR, hazard ratio; IPTW, inverse probability of treatment weighting.

**Table S2. Standardized Mean Differences Across Variables**

| Variables | Medication (n=1225) | TEVAR  (n=67) | SMD  (Crude) | SMD  (IPTW adjusted) |
| --- | --- | --- | --- | --- |
| Male | 741 (60.5) | 48 (71.6) | 0.237 | 0.067 |
| Age category |  |  | 0.453 | 0.022 |
| <50 | 28 (2.3) | 1 (1.4) |  |  |
| 50-59 | 44 (3.6) | 3 (4.5) |  |  |
| 60-69 | 190 (15.5) | 14 (20.9) |  |  |
| 70-79 | 368 (30.0) | 27 (40.3) |  |  |
| 80-89 | 470 (38.4) | 21 (31.3) |  |  |
| 90- | 125 (10.2) | 1 (1.5) |  |  |
| Year of admission |  |  | 0.237 | 0.093 |
| 2013-2014 | 251 (20.5) | 9 (13.4) |  |  |
| 2015-2016 | 351 (28.7) | 18 (26.9) |  |  |
| 2017-2018 | 355 (29.0) | 20 (29.9) |  |  |
| 2019-2020 | 268 (21.9) | 20 (29.9) |  |  |
| Area category |  |  | 0.155 | 0.109 |
| West | 420 (34.3) | 23 (34.3) |  |  |
| Middle | 401 (32.7) | 20 (29.9) |  |  |
| East | 363 (29.6) | 23 (34.3) |  |  |
| Other | 41 (3.4) | 1 (1.5) |  |  |
| Elixhauser Comorbidity |  |  |  |  |
| Heart failure | 276 (22.5) | 11 (16.4) | 0.155 | 0.007 |
| PAD | 238 (19.4) | 16 (23.9) | 0.108 | 0.005 |
| Pulmonary disease | 309 (25.2) | 20 (29.9) | 0.104 | 0.066 |
| Hemiplegia | 25 (2.0) | 0 | NA | NA |
| Weight loss | 3 (0.2) | 1 (1.5) | 0.135 | <0.001 |
| Fluid and electrolyte disorders | 199 (16.2) | 11 (16.4) | 0.005 | 0.026 |
| Anemia | 169 (13.8) | 7 (10.4) | 0.103 | 0.041 |
| Alcohol abuse | 4 (0.3) | 1 (1.5) | 0.123 | 0.001 |
| Depression | 73 (6.0) | 6 (9.0) | 0.114 | 0.061 |
| Psychoses | 42 (3.4) | 0 | NA | NA |
| Arrhythmias | 238 (19.4) | 11 (16.4) | 0.079 | 0.009 |
| Valvular disease | 86 (7.0) | 5 (7.5) | 0.017 | 0.033 |
| Pulmonary circulation disorders | 8 (0.7) | 1 (1.5) | 0.082 | 0.002 |
| Hypertension | 750 (61.2) | 41 (61.2) | 0.001 | 0.034 |
| Other neurological disorders | 64 (5.2) | 4 (6.0) | 0.032 | 0.019 |
| Diabetes | 66 (5.4) | 3 (4.5) | 0.016 | 0.025 |
| Hypothyroidism | 35 (2.9) | 4 (6.0) | 0.152 | 0.007 |
| Renal failure | 110 (9.0) | 10 (14.9) | 0.184 | 0.015 |
| Liver disease | 175 (14.3) | 9 (13.4) | 0.025 | 0.013 |
| Peptic ulcer disease | 253 (20.7) | 13 (19.4) | 0.031 | 0.048 |
| Lymphoma | 4 (0.3) | 1 (1.5) | 0.123 | 0.002 |
| Tumor | 151 (12.3) | 8 (11.9) | 0.019 | 0.008 |
| Rheumatoid arthritis / collagen vascular diseases | 74 (6.0) | 4 (6.0) | 0.003 | 0.031 |
| Coagulopathy | 33 (2.7) | 1 (1.5) | 0.084 | 0.023 |
| Obesity | 2 (0.2) | 1 (1.5) | 0.147 | <0.001 |
| History of Aortic Aneurysm | 138 (11.3) | 12 (17.9) | 0.189 | 0.030 |
| Previous TEVAR | 3 (0.2) | 1 ( 1.5) | 0.135 | <0.001 |

Categorical variables are presented as n (%). IPTW, inverse probability of treatment weighting; PAD, peripheral arterial disease; SMD, standardized mean difference; TEVAR, thoracic endovascular aortic repair.

**Table S3. Comparison of Standardized Mean Differences Across Models**

| Variables | SMD  (Crude) | SMD  (IPTW adjusted: Main) | SMD  (IPTW adjusted: Sensitivity) |
| --- | --- | --- | --- |
| Male | 0.237 | 0.067 | 0.080 |
| Age_category | 0.453 | 0.022 | 0.029 |
| Year of admission | 0.237 | 0.093 | 0.110 |
| Area category | 0.155 | 0.109 | 0.114 |
| Elixhauser Comorbidity |  |  |  |
| Heart failure | 0.155 | 0.007 | 0.008 |
| PAD | 0.108 | 0.005 | 0.006 |
| Pulmonary disease | 0.104 | 0.066 | 0.062 |
| Weight loss | 0.135 | <0.001 | <0.001 |
| Fluid and electrolyte disorders | 0.005 | 0.026 | 0.040 |
| Anemia | 0.103 | 0.041 | 0.045 |
| Alcohol abuse | 0.123 | 0.001 | <0.001 |
| Depression | 0.114 | 0.061 | 0.076 |
| Arrhythmias | 0.079 | 0.009 | 0.014 |
| Valvular disease | 0.017 | 0.033 | 0.035 |
| Pulmonary circulation disorders | 0.082 | 0.002 | 0.002 |
| Hypertension | 0.001 | 0.034 | 0.010 |
| Other neurological disorders | 0.032 | 0.019 | 0.009 |
| Diabetes | 0.016 | 0.025 | 0.026 |
| Hypothyroidism | 0.152 | 0.007 | 0.005 |
| Renal failure | 0.184 | 0.015 | 0.016 |
| Liver disease | 0.025 | 0.013 | 0.018 |
| Peptic ulcer disease | 0.031 | 0.048 | 0.057 |
| Lymphoma | 0.123 | 0.002 | 0.002 |
| Tumor | 0.019 | 0.008 | 0.014 |
| Rheumatoid arthritis / collagen vascular diseases | 0.003 | 0.031 | 0.030 |
| Coagulopathy | 0.084 | 0.023 | 0.023 |
| Obesity | 0.147 | <0.001 | <0.001 |
| History of Aortic Aneurysm | 0.189 | 0.030 | 0.032 |
| Previous TEVAR | 0.135 | <0.001 | <0.001 |

SMD (IPTW adjusted: Main): After adjustment using IPTW based on the main analysis model, which includes all variables. SMD (IPTW adjusted: Sensitivity): After adjustment using IPTW based on the sensitivity analysis model, where two low-frequency variables (hemiplegia and psychoses) were excluded. These variables were excluded because of their extremely low prevalence in the cohort and the absence of any cases in the treatment group. IPTW, inverse probability of treatment weighting; PAD, peripheral arterial disease; SMD, standardized mean difference; TEVAR, thoracic endovascular aortic repair.
