# Supplementary figures and images for: Real-world outcomes and management trends in uncomplicated type B aortic dissection
Source: Interdiscip Cardiovasc Thorac Surg. 2025 Apr 11;40(4):ivaf089. doi: 10.1093/icvts/ivaf089 (PMC12022216; doi:10.1093/icvts/ivaf089)

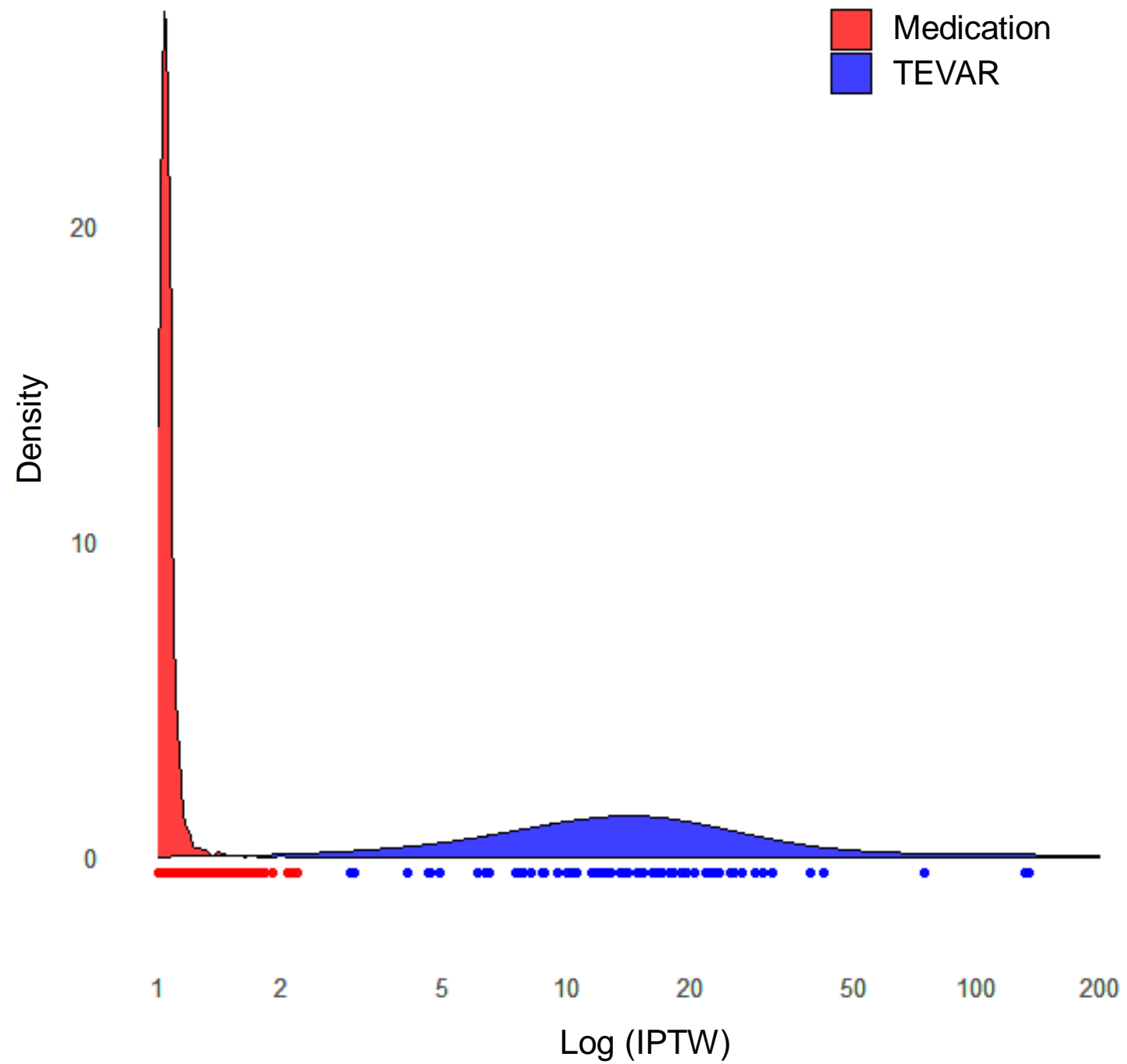

Supplement: ivaf089_Supplementary_Data [file ivaf089_supplementary_data.zip › ivaf089_Supplementary_Data/FigS1.pdf]

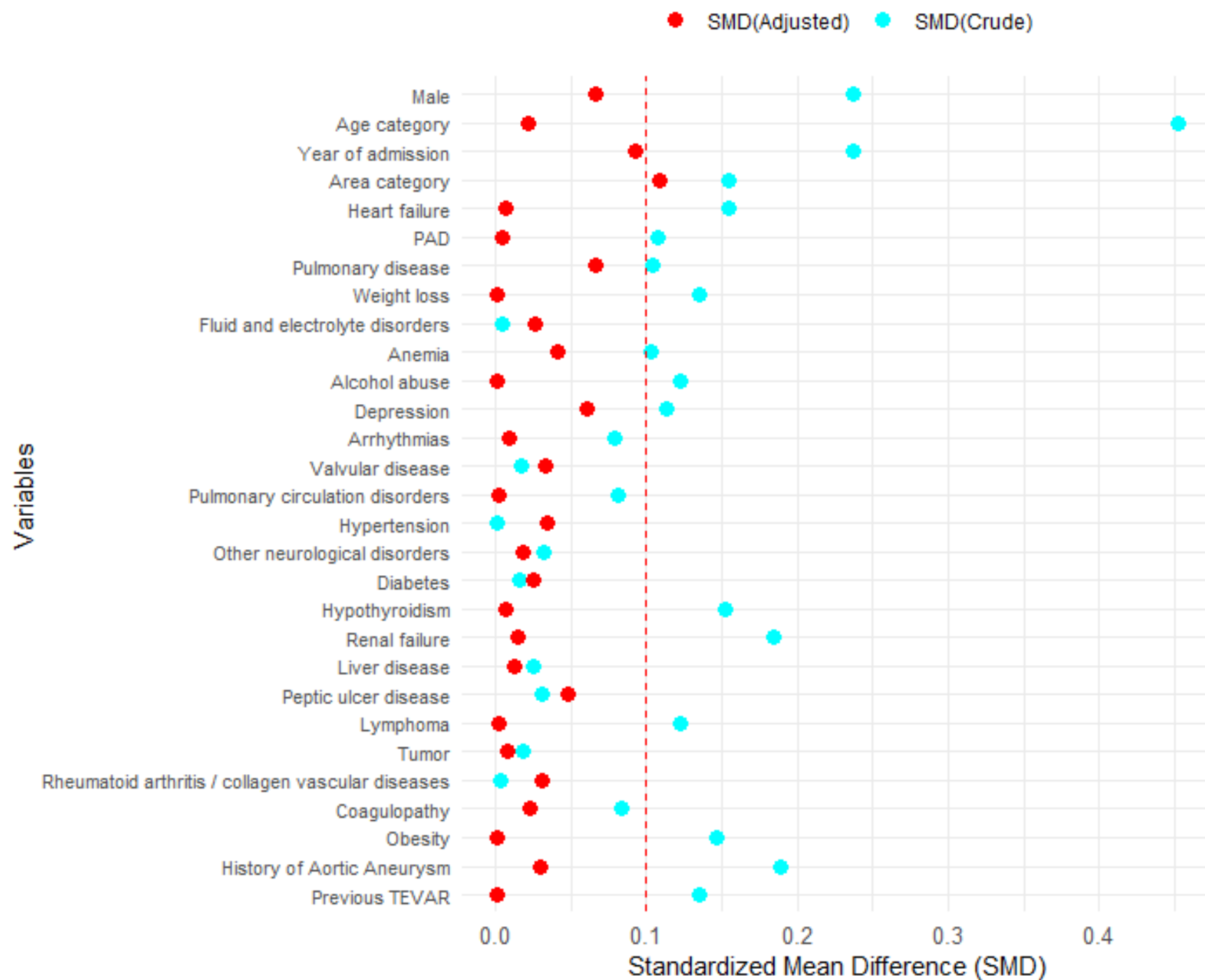

Supplement: ivaf089_Supplementary_Data [file ivaf089_supplementary_data.zip › ivaf089_Supplementary_Data/FigS2.pdf]
